# Prevalence of dental caries among children in Indonesia: A systematic review and meta-analysis of observational studies

**DOI:** 10.1016/j.heliyon.2024.e32102

**Published:** 2024-05-29

**Authors:** Faizul Hasan, Lia Taurussia Yuliana, Hendrik Setia Budi, Rajesh Ramasamy, Zilzala Irqon Ambiya, Anindya Marsa Ghaisani

**Affiliations:** a Faculty of Nursing, Chulalongkorn University, Bangkok, 10330, Thailand; b Department of Oral Biology, Dental Pharmacology, Faculty of Dental Medicine, Universitas Airlangga, Surabaya, 60132, Indonesia; c Immunology Unit, Department of Pathology, Faculty of Medicine and Health Sciences, Universiti Putra Malaysia, Selangor, 43400, Malaysia; d Department of Oral Biology, Faculty of Dental Medicine, Universitas Airlangga, Surabaya, 60132, Indonesia

**Keywords:** Dental caries, Indonesia, Systematic review, Capital-urban city, Child well-being

## Abstract

**Objective:**

The prevalence of dental caries among children in Indonesia remains unclear. Therefore, we aimed to provide an updated assessment of this prevalence while also investigating the influence of patient characteristics and methodological factors.

**Design:**

We performed a systematic review and meta-analysis, including searches of PubMed, Cochrane Library, and Embase from inception to August 24, 2023. We included 8840 participants in 27 studies reporting the prevalence of dental caries among Indonesian children.

**Results:**

The overall prevalence of dental caries was 76 % (95 % confidence interval: 71%–81 %). Studies in which decay-missing-filled teeth (DMFT) criteria were used to diagnose dental caries were significantly more prevalent than studies using non-DMFT criteria (78 % vs. 64 %, P < 0.05). No significant moderators were identified for the study subgroup based on study origin (Jakarta vs. non-Jakarta) or comorbidity status (comorbidity vs. no comorbidity). Owing to incomplete reporting of variables, metaregression analysis could not be conducted for continuous variables, such as age and male percentage.

**Conclusions:**

The prevalence of dental caries among Indonesian children remains notably high, showing consistency across Jakarta-based studies and non-Jakarta studies. Initiating dental caries prevention and health promotion campaigns is imperative, focusing on the critical importance of early detection.

## 1 Introduction

Dental caries, also known as tooth decay, is a global issue. It arises from the fermentation of dietary carbohydrates by bacteria, leading to the production of acidic byproducts that demineralize dental enamel [1]. Microbiome dysbiosis is a contributing factor, involving various acid-producing and -tolerant organisms, such as *Streptococcus mutans*, *Lactobacillus* spp., *Scardovia wiggsiae*, and *Actinomyces* spp [2,3]. In children, dental caries is associated with dental pain, discomfort during eating, potential tooth loss, and nutrition challenges due to eating difficulties. Additionally, it has linked to increased infection risks, abscess formation, implications for permanent teeth development, potential for dental anxiety, and even adverse effects on academic performance [4]. Hence, early detection of dental caries is crucial.

According to the 2018 Basic Health Research (Riskesdas) findings, the prevalence of dental and oral health problems in Indonesia was 57.6 % [5]. The decay-missing-filled teeth (DMFT) index, endorsed by the World Health Organization (WHO), serves as a critical oral health status indicator, facilitating the measurement and comparison of tooth decay rates [6,7]. This index evaluates decayed, missing, and filled teeth, thereby playing a pivotal role in analyzing, controlling, developing, and implementing oral health initiatives [8]. Children aged 5 years with a caries severity score of DMFT >6 fall into the severe early childhood caries category [4].

Globally, prevalence of dental caries varies widely among children. The WHO estimates that dental caries frequency in 5–6-year-old children from countries with low and intermediate incomes is 60%–90 %, whereas in high-income nations, 20%–40 % of children in this age group are affected [9]. Regional and demographic differences have prompted studies in various countries to assess dental caries prevalence. In East Africa, a systematic review and meta-analysis revealed a dental caries prevalence of 40.98 % [10]. In Ethiopia, 65.5 % of 3–5-year-olds were reported to have dental caries [11]. These findings underscore the substantial burden of dental caries in these specific demographics [12]. In Indonesia, updating and improving data on the prevalence and severity of dental caries in children is imperative to facilitate efficient oral health policy planning and resource allocation.

## 2 Materials and methods

### 2.1 Data sources and searches

This systematic review and meta-analysis adhered to the Preferred Reporting Items for Systematic Reviews and Meta-analysis guidelines. Registration for this study and meta-analysis was completed in PROSPERO (No. CRD42023457894). Various databases (PubMed, Cochrane Library, and Embase) were searched from inception until August 24, 2023. The keyword combinations used were as follows: “dental caries” OR “caries” OR “tooth decay” OR “DMFT index” OR “decayed teeth” OR “root caries” OR “cervical caries” OR “root surface caries” AND “Indonesia.” An example search strategy is provided in Suppl. Table 1. Additionally, all retrieved studies were searched manually to identify similar studies meeting the inclusion criteria.

### 2.2 Study selection

We included full-text prospective, cross-sectional, retrospective, and case control studies reporting dental caries prevalence in children, without including language restrictions. Titles and abstracts were independently reviewed and analyzed for eligibility by two reviewers (FH and LTY). Any discrepancies were resolved through discussion with a third investigator (HSB).

### 2.3 Data extraction and methodological quality assessment

Two reviewers (F.H and LTY) independently collected data and addressed inconsistencies. Extracted data included author, publication year, study origin and design, participant age, male percentage, body mass index (BMI), comorbidity status, diagnostic measurement, sample size, cases, and prevalence rate. Methodological quality was assessed using the critical appraisal checklist recommended by the Joanna Briggs Institute [13], with eight items, each rated as “yes,” “no,” “unclear,” or “not applicable,” used to determine the quality of cross-sectional studies. Analyses were also conducted independently by two reviewers (F.H and LTY), with any disputes settled through consensus meetings.

### 2.4 Outcome measure descriptions

The DMFT scale was predominantly used for detecting dental caries. Additionally, oral diagnostic tools, physical examinations, and saliva samples were used in 4 out of the 25 studies included.

### 2.5 Data synthesis and analysis

All analyses were performed using Comprehensive Meta-Analysis Software, 2.0 (Biostat, Englewood, NJ, USA). A random-effects model was used to estimate dental caries prevalence with 95 % confidence intervals (CIs). Heterogeneity among studies was evaluated using Cochran's Q and I^2^ tests, with values of <0.05 and 50 % indicating significant heterogeneity, respectively [14]. Subgroup analysis was conducted to determine potential sources of heterogeneity. Egger's intercept test was employed to assess publication bias, with P < 0.05 indicating statistical significance [14].

## 3 Results

### 3.1 Selection, inclusion, and characteristics of studies

**Fig. 1:**
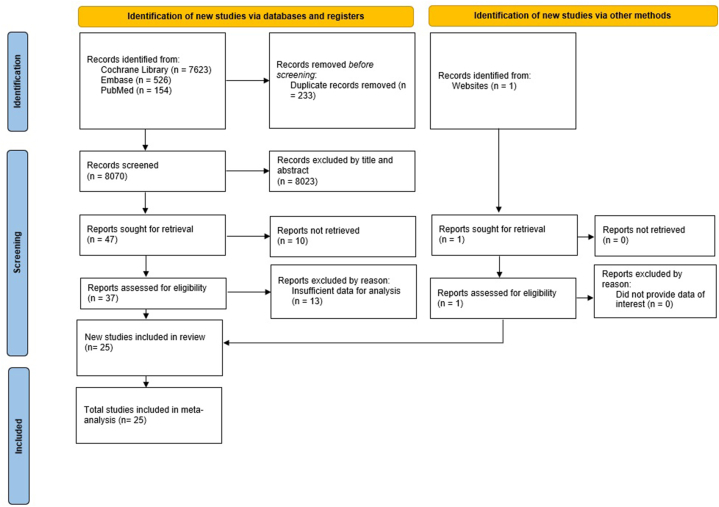
PRISMA 2020 flow diagram.

As shown in Fig. 1, we initially identified 8303 potentially eligible articles. Of these, 233 duplicates were removed. Following screening of abstracts and titles, 8023 records were excluded based on inclusion and exclusion criteria. In total, 37 full-text articles met the inclusion criteria and underwent further review. Of these, 13 studies were excluded, with the reasons detailed in Suppl. Table 2. Additionally, one study was sourced from website searching. Ultimately, 25 full-text articles meeting the inclusion criteria were retained [[15], [16], [17], [18], [19], [20], [21], [22], [23], [24], [25], [26], [27], [28], [29], [30], [31], [32], [33], [34], [35], [36], [37], [38], [39]]. These studies comprised a total sample size of 8840 participants. Among the 25 studies, 22 used the DMFT index for dental caries measurement, with most studies employing a cross-sectional study design. Detailed characteristics of the included studies are provided in Table 1.

**Table 1:** Study characteristic of dental caries.

First authors, year	City	Study design	Age	Male %	BMI	Sample size	Comorbidity	Tool	Cut point	n case	Prevalence %
Achmad, 2019 [15]	Makassar	Cross-sectional study	2–6 yr	45.4	Normal (67 %)	506	Y	Oral diagnostic tool	NR	353	69.8
					Overweight (13.8 %)						
					Underweight (11.1 %)						
					Obesity (8.1 %)						
Adiatman, 2016a [16]	Jakarta	Cross-sectional study	5 yr	52 %	NR	390	NR	DMFT	≥1	351	90
Adiatman, 2016b [16]	Jakarta	Cross-sectional study	12 yr	50 %	NR	458	NR	DMFT	≥1	385	84
Aldy, 1979 [17]	Medan	Observational study	<5 yr		NR	100	NR	Physical examination	NR	54	54
Aliyah, 2020 [18]	Surabaya	Cross-sectional study	12 yr	NR	NR	102	NR	Single permanent molar dental caries	4	76	74.5
Amalia, 2012 [19]	Yogjakarta	Cross-sectional study	12 yr	53.8	NR	1906	NR	DMFT	≥1	1533	80.43
Aziza, 2020 [20]	Surabaya	Cross-sectional study	3–6 yr	NR	NR	110	NR	DMFT	8.4	97	88
Bachtiar, 2018 [21]	Jakarta	Cross-sectional study	3–5 yr	NR	NR	32	NR	Saliva sample	NR	16	50
Badruddin, 2017 [22]	Jakarta	Cross-sectional study	30 mo	NR	NR	281	Y	DMFT	NR	155	55.2
Bramantoro, 2019 [23]	Surabaya	Cross-sectional study	<12 yr	50.4	NR	213	NR	DMFT	1	113	53
Budipramana, 2002 [24]	Surabaya	Cross-sectional study	6–12	NR	NR	474	NR	DMFT	NR	Permanent teeth caries (180)	Permanent teeth caries (38)
										Primary teeth caries (156)	Primary teeth caries (33)
Fauzia, 2020 [25]	Jakarta	Cross-sectional study	36–71 mo	NR	NR	165	NR	DMFT	NR	89	53.9
Hariyani, 2019 [26]	Surabaya	Cross-sectional study	Children ≤12 and > 13	74.3 %	NR	70	Autist	DMFT	NR	55	78.6
Khairinisa, 2023 [27]	Jakarta	Cross-sectional study	5 yr	50.4 %	NR	266	NR	DMFT	≥1	238	89.4
Koloway, 1992 [28]	Bandung, pangalengan	Cross-sectional study	4–5 yr	NR	NR	459	NR	DMFT	NR	428	93.2
Lendrawati, 2019 [29]	Padang	Cross-sectional study	12–15 yr	42 %	NR	150	NR	DMFT	≥1	92	61
Maharani, 2017a [30]	Bekasi	Cross-sectional study	6–7 yr	46 %	NR	539	NR	DMFT	NR	507	94
Maharani, 2017b [30]	Bekasi	Cross-sectional study	10–11 yr	42 %	NR	445	NR	DMFT	NR	401	90
Maharani, 2019a [31]	Jakarta	Cross-sectional study	12–15 yr	41.5 %	NR	494	NR	DMFT	≥1	343	69.4
Maharani, 2019b [32]	Jakarta	Cross-sectional study	12 yr	43 %	NR	696	NR	DMFT	≥1	425	61
Nugraha, 2020 [33]	Surabaya	Cross-sectional study	1–12 yr	51.7 %	NR	29	Children Living with Perinatal HIV/AIDS (CLWPHA)	DMFT	NR	25	86.2
Rachmawati, 2019 [34]	Jakarta	Cross-sectional study	12–13 yr	50.4 %	NR	341	NR	DMFT	≥1	252	74
Rachmawati, 2017 [35]	Jakarta	Cross-sectional study	5–6 yr	50.3 %	NR	161	NR	DMFT	≥1	132	82
Ramadhani, 2021 [36]	Jakarta	Cross-sectional study	5 yr	50.4 %	NR	266	NR	DMFT	≥1	236	88.7
Rieuwpassa, 2019 [37]	Raja ampat	Cross-sectional study	8–13 yr	42 %	NR	50	NR	DMFT	≥1	10	20
Setiawan, 2020 [38]	Bandung	Cross-sectional study	2–3 yr	48.3 %	NR	87	NR	DMFT	≥1	62	71.3
Susilo, 2020 [39]	Madura	Cross-sectional study	3–6 yr	NR	NR	50	NR	DMFT	≥1	43	86

AIDS= Acquired Immunodeficiency Syndrome; BMI= Body Mass Index; DMFT = Decayed, Missing and Filled Teeth; HIV= Human Immunodeficiency Virus; mo = Month; n = Number of participants; NR= Not reported; yr = Year.

### 3.2 Prevalence of dental caries

**Fig. 2:**
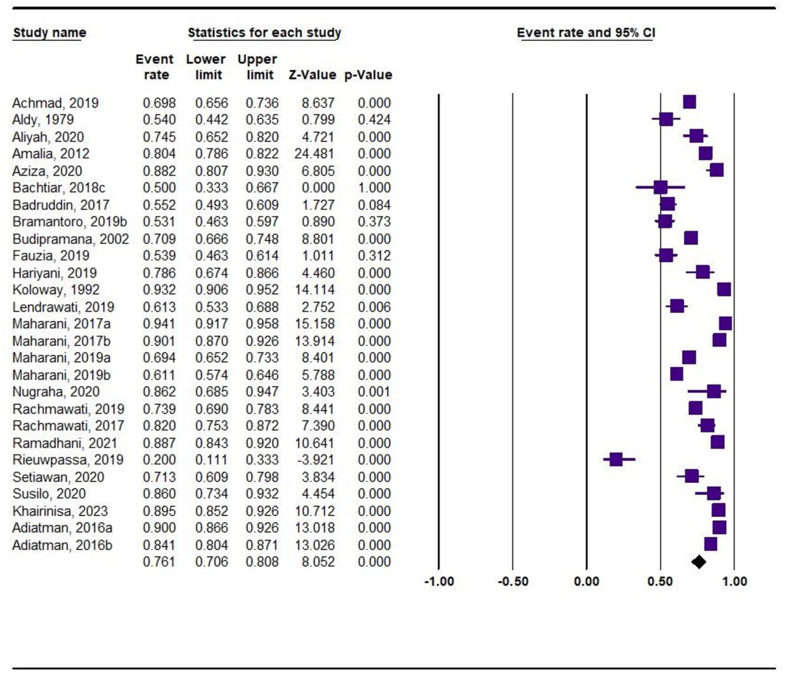
Forest plot of overall dental caries prevalence. CI: confidence interval.

The prevalence of dental caries among the included studies is summarized in Fig. 2. The overall prevalence was 76 % (95 % CI: 71%–81 %). Substantial heterogeneity was observed among the included studies (I^2^: 96 %; P < 0.001).

### 3.3 Subgroup analyses

**Fig. 3:**
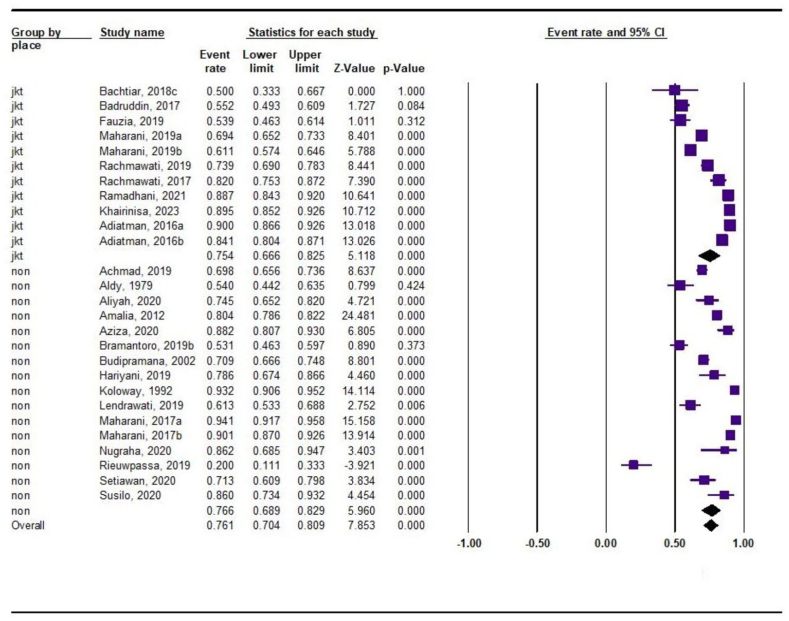
Forest plot of dental caries subgroups based on study location. CI: confidence interval; jkt: Jakarta; non: non-Jakarta.

The subgroup comparison between studies based on DMFT diagnostic criteria vs. non-DMFT criteria is presented in Suppl. Fig. 1. Studies employing DMFT criteria for diagnosing dental caries exhibited significantly higher prevalence compared with those using non-DMFT criteria (78 % vs. 64 %, P < 0.05). Additionally, dental caries prevalence in Jakarta-based studies was comparable to that in non-Jakarta studies (75.4 % vs. 76.6 %, P > 0.05; Fig. 3). Similarly, no significant differences were found between studies grouped based on comorbidity status (comorbidity vs. no comorbidity; Suppl. Fig. 2).

### 3.4 Methodology quality assessment of included studies

Critical appraisal checklist results for the included studies are presented in Suppl. Table 3. According to the Joanna Briggs Institute's recommended critical appraisal checklist for cross-sectional studies, comprising eight items, all 27 included studies were rated “Yes” for items 1–4. For items 5 and 6, 12 out of 27 studies were rated “Yes,” whereas the others were rated “Unclear.” For items 7 and 8, 26 out of 27 studies were rated “Yes,” with 1 study rated “Unclear.”

### 3.5 Publication bias

No evidence of publication bias was found based on Begg's and Egger's test criteria (all P > 0.05).

## 4 Discussion

To the best of our knowledge, this systematic review and meta-analysis represent the first comprehensive investigation into the prevalence of dental caries among children in Indonesia. Our findings reveal a persistently high prevalence of caries, with similar rates observed across sample locations (Jakarta vs. non-Jakarta). Given the rigorous methodology employed in this study, these findings warrant substantial consideration.

Principal oral bacteria include both gram-positive and gram-negative species, along with aerobic and anaerobic strains. Changes in the microenvironment can disrupt the commensal flora–host interaction, leading to disease. Dysbiotic microbiota contribute to the development of caries and periodontal diseases [40]. Pathogenic bacteria, especially those forming biofilms, counteract beneficial bacteria action. Oral biofilm polymicrobial composition includes *Streptococcus*, *Actinomyces*, *Lactobacillus*, *Veillonella* [41], *Neisseria*, and *Eubacterium* [42,43]. Although biofilms can thrive under human homeostasis, they are also responsible for tooth caries and periodontitis, the two most prevalent oral disorders [44,45].

Caries was found in >50 % of research subjects aged <11 years, with qualifying age categories including toddlers (1–5 years), children (6–10 years), and adolescents (10–19 years) [4]. Cognitive abilities and analytical capacities tend to increase with age, enabling individuals to acquire more knowledge. Indeed, a correlation exists between age and an individual's knowledge level, with older individuals displaying greater maturity and mental acuity compared with younger individuals [46,47]. In addition to age, both education and occupation can influence an individual's knowledge level [48]. Education markedly influences knowledge accumulation, as higher education levels correlate with enhanced information processing and knowledge accumulation [49]. Furthermore, age encompasses intrinsic factors, such as experience, environment, and prior knowledge, contributing to an individual's understanding of health maintenance [50].

Unhealthy dietary habits, including consumption of high-calorie food, contribute to increased caries prevalence [51]. Additionally, dietary factors have contributed to the global rise in the rates of overweight children and childhood obesity in recent decades. Studies exploring the association between dental caries and excess weight/obesity in children have highlighted shared risk factors [52,53]. We found that males and females in the lower BMI categories face a higher risk of caries development, consistent with the findings of extensive surveys conducted in Guangzhou [54] and the United States [55]. Severe caries often leads to toothache, reducing children's ability and willingness to eat, thereby diminishing their food intake [55,56].

Certain children may experience concurrent medical and mental health conditions, such as epilepsy and anxiety, respectively, akin to attention deficit/hyperactivity disorder, a neuropsychiatric condition impacting brain functioning. Symptoms manifest in an individual's energetic demeanor, concentration issues, and engagement in impulsive behaviors. Moreover, mental health–related symptoms may contribute to malnutrition, subsequently affecting dental health. Individuals with mental illness, especially those with severe symptoms, may struggle to adhere to consistent daily dental care routines and access necessary dental treatment [57].

Regarding caries severity assessment methods, 22 out of 27 studies used the DMFT index, whereas the remaining studies did not. The DMFT index, recommended by the WHO, measures caries prevalence based on cavitated caries lesions, making it the most commonly used dental caries index in epidemiological studies [58]. Early diagnosis of noncavitated carious lesions enables preventive measures, potentially preventing caries-related morbidity and reducing the financial burden associated with restorative or rehabilitative dental care. The International Caries Detection and Assessment System (ICDAS II) incorporates both cavitated and noncavitated carious lesions with acceptable reliability [59,60].

Finally, in interpreting our findings, several limitations must be acknowledged. First, variations in participant characteristics and study details, including comorbidities, caries diagnosis criteria, and sample size, contribute to study heterogeneity. Second, owing to incomplete reporting of continuous variables, such as age and male percentage, in some studies, metaregression analysis could not be performed. Nevertheless, the use of rigorous study methods and a substantial sample size enhance the credibility of this meta-analysis.

## 5 Conclusions

Our study highlights the persistence of high dental caries prevalence among Indonesian children, with consistent rates observed across urban and nonurban areas. Urgent implementation of dental caries prevention health promotion campaigns is warranted, alongside prioritizing early screening for dental caries.

## Data availability statement

Data included in article/supp. material/referenced in article:

## CRediT authorship contribution statement

**Faizul Hasan:** Writing – original draft, Visualization, Validation, Software, Formal analysis, Conceptualization. **Lia Taurussia Yuliana:** Writing – original draft, Visualization, Validation, Software, Formal analysis, Conceptualization. **Hendrik Setia Budi:** Writing – review & editing, Writing – original draft, Supervision, Resources, Methodology, Investigation, Funding acquisition, Formal analysis, Data curation, Conceptualization. **Rajesh Ramasamy:** Writing – original draft, Validation. **Zilzala Irqon Ambiya:** Project administration, Investigation. **Anindya Marsa Ghaisani:** Project administration, Investigation.

## Declaration of competing interest

The authors declare the following financial interests/personal relationships which may be considered as potential competing interests: Hendrik Setia Budi reports article publishing charges was provided by Airlangga University. If there are other authors, they declare that they have no known competing financial interests or personal relationships that could have appeared to influence the work reported in this paper.
