# Supplementary material for: Prevalence of dental caries among children in Indonesia: A systematic review and meta-analysis of observational studies
Source: Heliyon. 2024 May 29;10(11):e32102. doi: 10.1016/j.heliyon.2024.e32102 (PMC11176858; doi:10.1016/j.heliyon.2024.e32102)
Supplement: Multimedia component 1 [file mmc1.docx]

**Supplementary Table 2**

Study excluded by reason.

| First authors, year | Reason |
| --- | --- |
| Adiningrat, 2020 | Insufficiency data |
| Bachtiar, 2018a | Insufficiency data |
| Bachtiar, 2018b | Insufficiency data |
| Bramantoro, 2020 | Insufficiency data |
| De soet 2003 | Insufficiency data |
| Dewanto, 2020 | Insufficiency data |
| Dimaisip-Nabuab, 2018 | Insufficiency data |
| Laksmiastuti, 2019 | Insufficiency data |
| Laksmiastuti, 2017 | Insufficiency data |
| Mislia, 2020 | Insufficiency data |
| Ngatemi | Insufficiency data |
| Permatasari, 2020 | Insufficiency data |
| Re, 2021 | Insufficiency data |
