# Supplementary material for: Prevalence of dental caries among children in Indonesia: A systematic review and meta-analysis of observational studies
Source: Heliyon. 2024 May 29;10(11):e32102. doi: 10.1016/j.heliyon.2024.e32102 (PMC11176858; doi:10.1016/j.heliyon.2024.e32102)
Supplement: Multimedia component 2 [file mmc2.docx]

**Supplementary Table 1**

Search strategy.

| Database | Keywords |
| --- | --- |
| PubMed | ("Dental Caries"[All Fields] OR ("caries"[All Fields] OR "Dental Caries"[MeSH Terms] OR ("dental"[All Fields] AND "caries"[All Fields]) OR "Dental Caries"[All Fields] OR "caries"[All Fields]) OR "Tooth decay"[All Fields] OR "DMFT index"[All Fields] OR "decayed teeth"[All Fields] OR "root caries"[All Fields] OR "cervical caries"[All Fields] OR "root surface caries"[All Fields]) AND ("Indonesia"[MeSH Terms] OR "Indonesia"[All Fields] OR "Indonesia s"[All Fields] OR "Indonesias"[All Fields]) |
| Cochrane library | "Dental Caries" OR caries OR "Tooth decay" OR "DMFT index" OR "decayed teeth" OR "root caries" OR "cervical caries" OR "root surface caries" AND Indonesia |
| Embase | "Dental Caries" OR caries OR "Tooth decay" OR "DMFT index" OR "decayed teeth" OR "root caries" OR "cervical caries" OR "root surface caries" AND Indonesia |
