# Supplementary material for: Prevalence of dental caries among children in Indonesia: A systematic review and meta-analysis of observational studies
Source: Heliyon. 2024 May 29;10(11):e32102. doi: 10.1016/j.heliyon.2024.e32102 (PMC11176858; doi:10.1016/j.heliyon.2024.e32102)
Supplement: Multimedia component 3 [file mmc3.docx]

**Suppl. Fig. 1.** Forest plot of dental caries subgroup by diagnostic tool. CI= confidence interval; DMFT= The Decayed, Missing and Filled Teeth index; non= non DMFT.
