# Supplementary material for: Prevalence of dental caries among children in Indonesia: A systematic review and meta-analysis of observational studies
Source: Heliyon. 2024 May 29;10(11):e32102. doi: 10.1016/j.heliyon.2024.e32102 (PMC11176858; doi:10.1016/j.heliyon.2024.e32102)
Supplement: Multimedia component 4 [file mmc4.docx]

**Suppl. Fig. 2.** Forest plot of dental caries subgroup by comorbidity. CI= confidence interval; NR= not reported; Y= yes.
