# Supplementary material for: Prevalence of dental caries among children in Indonesia: A systematic review and meta-analysis of observational studies
Source: Heliyon. 2024 May 29;10(11):e32102. doi: 10.1016/j.heliyon.2024.e32102 (PMC11176858; doi:10.1016/j.heliyon.2024.e32102)
Supplement: Multimedia component 5 [file mmc5.docx]

**Supplementary Table 3**

Quality assessment of included studies.

| First authors, year | Q1 | Q2 | Q3 | Q4 | Q5 | Q6 | Q7 | Q8 |
| --- | --- | --- | --- | --- | --- | --- | --- | --- |
| Achmad, 2019 [15] | Y | Y | Y | Y | Y | Y | Y | Y |
| Adiatman, 2016a [16] | Y | Y | Y | Y | U | U | Y | Y |
| Adiatman, 2016b [16] | Y | Y | Y | Y | U | U | Y | Y |
| Aldy, 1979 [17] | Y | Y | Y | Y | U | U | U | U |
| Aliyah, 2020 [18] | Y | Y | Y | Y | U | U | Y | Y |
| Amalia, 2012 [19] | Y | Y | Y | Y | U | U | Y | Y |
| Aziza, 2020 [20] | Y | Y | Y | Y | U | U | Y | Y |
| Bachtiar, 2018 [21] | Y | Y | Y | Y | U | U | Y | Y |
| Badruddin, 2017 [22] | Y | Y | Y | Y | U | U | Y | Y |
| Bramantoro, 2019 [23] | Y | Y | Y | Y | Y | Y | Y | Y |
| Budipramana, 2002 [24] | Y | Y | Y | Y | U | U | Y | Y |
| Fauzia, 2019 [25] | Y | Y | Y | Y | Y | Y | Y | Y |
| Hariyani, 2019 [26] | Y | Y | Y | Y | Y | Y | Y | Y |
| Khairinisa, 2023 [27] | Y | Y | Y | Y | Y | Y | Y | Y |
| Koloway, 1992 [28] | Y | Y | Y | Y | U | U | Y | Y |
| Lendrawati, 2019 [29] | Y | Y | Y | Y | Y | Y | Y | Y |
| Maharani, 2017a [30] | Y | Y | Y | Y | Y | Y | Y | Y |
| Maharani, 2017b [30] | Y | Y | Y | Y | Y | Y | Y | Y |
| Maharani, 2019a [31] | Y | Y | Y | Y | U | U | Y | Y |
| Maharani, 2019b [32] | Y | Y | Y | Y | U | U | Y | Y |
| Nugraha, 2020 [33] | Y | Y | Y | Y | U | U | Y | Y |
| Rachmawati, 2019 [34] | Y | Y | Y | Y | Y | Y | Y | Y |
| Rachmawati, 2017 [35] | Y | Y | Y | Y | Y | Y | Y | Y |
| Ramadhani, 2021 [36] | Y | Y | Y | Y | Y | Y | Y | Y |
| Rieuwpassa, 2019 [37] | Y | Y | Y | Y | U | U | Y | Y |
| Setiawan, 2020 [38] | Y | Y | Y | Y | Y | Y | Y | Y |
| Susilo, 2020 [39] | Y | Y | Y | Y | U | U | Y | Y |

N= no; NA= not applicable; Q= questions; U= unclear; Y= yes.

1. Were the criteria for inclusion in the sample clearly defined?
2. Were the study subjects and the setting described in detail?
3. Was the exposure measured in a valid and reliable way?
4. Were objective, standard criteria used for measurement of the condition?
5. Were confounding factors identified?
6. Were strategies to deal with confounding factors stated?
7. Were the outcomes measured in a valid and reliable way?
8. Was appropriate statistical analysis used?
